# Source Localization of Audiovisual Multisensory Neural Generators in Young Adults with Attention-Deficit/Hyperactivity Disorder

**DOI:** 10.3390/brainsci12060809

**Published:** 2022-06-20

**Authors:** Heather S. McCracken, Bernadette A. Murphy, Ushani Ambalavanar, Cheryl M. Glazebrook, Paul C. Yielder

**Affiliations:** 1 Faculty of Health Sciences, University of Ontario Institute of Technology, Oshawa, ON L1G 0C5, Canada; heather.mccracken@ontariotechu.net (H.S.M.); ushani.ambalavanar@ontariotechu.net (U.A.); paul.yielder@ontariotechu.ca (P.C.Y.); 2 Faculty of Kinesiology and Recreation Management, University of Manitoba, Winnipeg, MB R3T 2N2, Canada; cheryl.glazebrook@umanitoba.ca; 3 Health, Leisure and Human Performance Institute, University of Manitoba, Winnipeg, MB R3T 2N2, Canada; 4 School of Medicine, Deakin University, Geelong, VIC 3220, Australia

**Keywords:** standardised low-resolution brain electromagnetic tomography (sLORETA), source-localization, multisensory, Attention-Deficit/Hyperactivity Disorder (ADHD), Brodmann area (BA) 2, audiovisual

## Abstract

Attention-Deficit/Hyperactivity Disorder (ADHD) is a neurodevelopmental disorder that exhibits unique neurological and behavioural characteristics. Our previous work using event-related potentials demonstrated that adults with ADHD process audiovisual multisensory stimuli somewhat differently than neurotypical controls. This study utilised an audiovisual multisensory two-alternative forced-choice discrimination task. Continuous whole-head electroencephalography (EEG) was recorded. Source localization (sLORETA) software was utilised to determine differences in the contribution made by sources of neural generators pertinent to audiovisual multisensory processing in those with ADHD versus neurotypical controls. Source localization techniques elucidated that the controls had greater neural activity 164 ms post-stimulus onset when compared to the ADHD group, but only when responding to audiovisual stimuli. The source of the increased activity was found to be Brodmann Area 2, postcentral gyrus, right-hemispheric parietal lobe referenced to Montreal Neurological Institute (MNI) coordinates of X = 35, Y = −40, and Z = 70 (*p* < 0.05). No group differences were present during either of the unisensory conditions. Differences in the integration areas, particularly in the right-hemispheric parietal brain regions, were found in those with ADHD. These alterations may correspond to impaired attentional capabilities when presented with multiple simultaneous sensory inputs, as is the case during a multisensory condition.

## 1. Introduction

Attention-Deficit/Hyperactivity Disorder (ADHD) is a common neurodevelopmental disorder that is defined by specific behavioural characteristics [1]. Prominent behavioural characteristics of ADHD include hyperactivity, impulsivity, and inattention [2]. Although the most common signs and symptoms relating to ADHD are behavioural in nature, the literature has more recently suggested that there may be important neural alterations, with further research necessary to elucidate their relevance to ADHD symptomology. In the United States alone, it is estimated that 11% of children will receive a diagnosis of ADHD [2]. In addition to this, approximately 65% of children diagnosed with ADHD will continue to exhibit symptoms as adults [3]. Each of the hallmark behavioural signs can have important implications for many day-to-day activities that can hinder physical and mental health in adults with ADHD, thus resulting in both internal and external life stressors [4]. Therefore, a further understanding of these characteristics and their relevance to daily life is important. When comparing characteristics of ADHD in adulthood, adults are often noted as exhibiting reduced hyperactive tendencies when compared to children [5]. The less overt and disruptive presentation in adulthood may, in part, explain why literature tends to address the effects in childhood. Nevertheless, there is increasing recognition and awareness of ADHD in adulthood. Improving the understanding of behavioural and neurophysiological mechanisms prominent in adult ADHD is key, and this will enable further enhancements to environments and supports to assist and promote barrier-free function in this population.

There are well noted neural alterations associated with ADHD, relating to both functional and structural characteristics that are unique to this population. This includes diffuse reductions in grey matter found throughout the cortex, including parietal, temporal, frontal, and occipital brain regions [6,7,8,9,10]. Although many studies address such characteristics in children, allowing for an elaborate understanding of the neural substrates underlying childhood ADHD, persistent alterations are present in adults with ADHD as well and have recently become a focus of assessment [9,10]. In addition to diffuse grey matter alterations, characteristic alterations are present within the prefrontal cortex and related neural circuits [11]. Alterations within the prefrontal cortex are some of the most commonly described neurophysiological characteristics of ADHD. These structural changes may have important implications for the behavioural characteristics associated with ADHD, including alterations to executive functions, which are commonly associated with activity in prefrontal brain regions [12]. Notably, structural alterations in the prefrontal cortex were evident in adults [13]. Additionally, adolescents with ADHD have noted alterations to the insula, specifically the right anterior insula, as those with ADHD exhibit increased thickness when compared to neurotypical controls [8]. The insula, specifically Brodmann’s area (BA) 13, is involved in multisensory processes, likely those associated with stimulus identification [8,14]. This, in addition to parietal alterations, suggests that multisensory processing may be implicated in ADHD as a result of structural alterations, including, but not limited to, those related to the insula.

The neural substrates that underly attention and multisensory processing share many commonalities, including the superior colliculus, fronto-parietal, and temporo-parietal networks [15]. This, therefore, further suggests that those with attentional alterations may have a predisposition for altered multisensory processing. The capacity for multiple sensory inputs to be processed and integrated can have profound effects on behaviour and function. For instance, when multiple stimuli are presented simultaneously and integrated by the nervous system, this can speed response times and result in neural enhancements that can be quantified [16,17,18]. There are various forms of multisensory processing that may be imperative to day-to-day scenarios. A specific type of multisensory processing that is common to many everyday environments is audiovisual (AV) multisensory integration (MSI). AV integration occurs when an auditory and visual stimulus that are semantically congruent are presented closely in time and space. There are certain populations, such as those with autism spectrum disorder (ASD), that are thought to have altered AV MSI, potentially associated with difficulties in communication and social settings, where AV stimuli are abundant [19,20]. This illustrates the importance of MSI and how alterations to this process can have clinically and functionally significant implications. Due to the unique neural characteristics associated with ADHD, it is possible that alterations are also present in the way individuals with ADHD process multisensory inputs.

MSI is a form of neural processing that describes how the nervous system combines incoming sensory information, integrating this information in order to create a coherent perception of the surrounding environment [21]. Although ADHD is commonly associated with sensory-processing difficulties, limited literature has assessed multisensory processing in ADHD [22]. Of the work that has assessed multisensory processing in those with ADHD, it was noted that there was hypoactivation within BA 32 in the right brain hemisphere, which was evident when compared to the neurotypical controls, and this activation was associated with incongruent multisensory inputs [23]. One study utilised behavioural markers to analyse the temporal perception of multisensory input in those with ADHD-like traits and found that those with greater ADHD-like traits perceived fewer stimuli to be simultaneous than those with fewer ADHD-like traits [24]. This potentially affects the ability to integrate simultaneously occurring stimuli as multisensory due to distractibility. Recent work has suggested that adults with ADHD preferentially orient to auditory input during audiovisual conditions, and successful stimuli integration was associated with increased response time when compared to controls, thus suggesting that ADHD is associated with a reliance on auditory input when integrating AV stimuli [25]. Furthermore, this preferential reliance on auditory input may be correlated to hyperconnectivity between the planum temporale and the insula in adults with ADHD [25], as such neural substrates are associated with the processing of complex auditory input. A paper recently published in 2022 looked at multisensory processing in adult ADHD and found similar multisensory processing in those with ADHD when compared to controls [26]. Although, notably, those in the ADHD group exhibited race model violation for low-load conditions and not high-load conditions, which may suggest altered perceptual decision-making in situations that require high levels of attention [26]. Source localization has recently been used as an important tool to form an improved understanding of the neural characteristics associated with ADHD. Although indirectly related to multisensory processing, source imaging has identified an N2 modulation specific to successful inhibition in children with ADHD, with reduced activation localized to the right inferior frontal gyrus, supplemental motor area, and right temporoparietal junction [27]; whereas the successful inhibition of P3 was localized to reduced activation within the anterior cingulate cortex and supplementary motor area [27]. The unique differences in those with ADHD when processing unisensory and multisensory input may be a result of both behavioural and neurophysiological characteristics.

MSI is associated with functioning in specific brain regions and is thought to occur in the early phases of stimulus processing. The literature suggests that the integration of audiovisual multisensory inputs occurs prior to 200–250 ms after stimulus onset [28]. For instance, seminal research assessing the neural mechanisms associated with AV MSI via EEG found effects that were present over visual cortices as early as 40–90 ms, 110–145 ms, and 155–200 ms post-stimulus; in auditory cortices between 90 and 110 ms post-stimulus; and over right-hemispheric fronto-temporal brain regions between 140 and 165 ms [28]. Early multisensory work using EEG found significant multisensory neural enhancement over occipital brain regions at early processing latencies (~50 ms) [28,29], while Molholm and colleagues established similar interactive effects at approximately 46 ms residing over the visual cortex [30]. These analyses were performed based on the principle of superposition of electrical fields and thus limited to a surface assessment of electrical potentials, similar to that of our previous work [31,32]. Later integrative effects have been noted over the visual cortex in the time frame of 170 ms post-stimulus [33]. This is thought to be reflective of feedback modulation from higher-order multisensory areas [33]. fMRI imaging studies have suggested the presence of activation within auditory cortices when individuals are presented with visual input associated with linguistics, such as lipreading, even in conditions lacking auditory input [34]. This activation was specifically localized to primary auditory and auditory-associated cortices [34]. When comparing silent lipreading with conditions where auditory input was present, the lateral temporal auditory cortex exhibited similar activation, suggesting that underlying neural substrates are common to both seen and heard speech cues [34]. Such neural activity within auditory cortices is absent when presented with visual input that is not contextually relevant to auditory or speech processing [34]. This may suggest the important integrative effect that visual input can have on auditory conditions, particularly those that occur during speech-processing, allowing for crossmodal enhancements. Thus, AV stimuli provide an important opportunity to further understand multisensory processing, with relevance to many day-to-day tasks.

Neuroimaging techniques have illustrated underlying neural substrates that are imperative to many forms of sensory processing, including audition and vision, and those involved in the integration of such inputs for audiovisual processing [35]. Certain neural regions are involved in processing individual components of a multisensory input, whereas early research has described others as “multisensory”, such as parietal [36], temporal [37], and frontal areas [35,38]. In particular, research utilising EEG has found that cortical structures, including those found within parietal regions, are highly involved in the integration of audiovisual multisensory inputs [18,19,39,40]. Cortical regions, including the superior temporal sulcus and posterior parietal cortex, are two such multisensory regions [30]. These regions have a high density of multisensory neurons, being neurons that respond to both auditory and visual inputs [30,39]. The inferior parietal sulcus (IPS) and superior parietal lobule (SPL) are specific parietal regions to which multisensory inputs have been localized [39,41,42]. This suggests that parietal regions are heavily involved in AV MSI. In humans, AV multisensory processing is highly involved during speech and language perception. This interplay between auditory and visual input during speech can be exemplified by fMRI work showing activation within the auditory cortex during silent lipreading [34,40]. Similar methodologies utilising fMRI illustrate neural regions where super-additive responses occur to multisensory input, including the intra-parietal sulcus, insula, superior colliculus, ventral and dorsal prefrontal cortex, and the superior temporal sulcus [41]. Previous research addressing structures and sources implicated in multisensory processing can aid our interpretation of similar processes in various populations. The fact that many are cortical regions, as opposed to subcortical regions allows for the ability to assess them using techniques such as EEG.

Our previous research found that there were statistically significant and unique differences in how young adults with ADHD process AV multisensory inputs when compared to neurotypical controls [31,32]. These findings were based upon EEG event-related potential (ERP) data and noted several differences when compared to neurotypical controls [31]. For example, ADHD was associated with significantly enhanced neural processing of multisensory inputs in the parieto-occipital brain regions from 110 to 130 ms after stimulus onset. Behaviourally, those with ADHD had significantly faster response times, and although no significant accuracy differences were found, a medium effect size suggested increased error may be associated with ADHD and create a venue for future research to address [31]. It is possible that the unique neural response in those with ADHD may be related to the quicker responses in that study. In order to further develop an understanding of how ADHD and potential neural alterations present in this population, identifying specific neural generators related to important sensory and multisensory processes is necessary to highlight the role of specific neural regions that may function differently in ADHD.

Our previous work assessing MSI in ADHD involved the analysis of ERPs using super-additive models [31,32]; the results from this work were discussed above. This method utilised the audiovisual multisensory and unisensory high-density EEG waveforms to compare neural activity during multisensory responses [42]. Although this previous work provided novel and important information on multisensory processing in adults with ADHD, further incorporating a form of neural assessment that exceeds an assessment involving strictly surface electrical activity will provide insight into the role of specific cortical structures involved in MSI in adults with ADHD. These analyses were outside the scope of a strictly surface activity EEG assessment. Source localization is an analysis technique that pairs improved spatial resolution, due to the application of a standardised magnetic resonance imaging (MRI) template, with the temporal resolution of collected EEG data. It is clear that those with ADHD exhibit unique neural characteristics, and therefore assessing the neurophysiological response to stimuli using an analysis technique that provides enhanced spatial resolution is important. The current analysis source localized the evoked potential data from McCracken et al., 2020 [31]. For the present study, standardised low-resolution brain electromagnetic tomography (sLORETA) was used to localize neural activity recorded using 64-electrode whole-head EEG. sLORETA is a linear inverse algorithm that provides an estimate of the 3D distribution of neural generators within the cortex [43]. sLORETA has been found to provide the lowest localization error in comparison to other techniques using a linear inverse algorithm [43]. Therefore, source localization acts as an important and cost-effective method to assess neural substrates and their function in diverse populations.

The research question that this work aims to answer is whether there are specific neural structures involved in the processing of multisensory inputs in adults with ADHD that differ from neurotypical controls? It is hypothesised that there will be differences in which cortical regions are primarily involved in sensory processing, particularly those involved in MSI, such as the parietal brain regions.

## 2. Materials and Methods

### 2.1. Participants

This research received approval from the Ontario Tech University Research Ethics Board (REB), and participants provided written informed consent prior to participation. This study was performed according to the principles set out by the Declaration of Helsinki for the use of humans in experimental research. The participants included in the present analysis are identical to those whose multisensory ERP EEG peaks were assessed in McCracken et al., 2020 [31]. The participants were recruited from the Ontario Tech University student body and were contacted via in-course announcements and posters placed throughout the campus. The participants were young adults (18–35 years old) with ADHD, while the control group consisted of neurotypical adults with an absence of any known neurological conditions. The participants in the ADHD group had previously received an ADHD diagnosis from a registered health care professional. They self-reported the age at which they were diagnosed and also any medication that they typically took to manage their symptoms of ADHD at the time of participation. The ADHD group (*n* = 10, three females) had a mean age of 23.7 ± 3.3 years, with a mean age of diagnosis being 13.7 ± 7.7 years old. The neurotypical control group (*n* = 12, four females) had a mean age of 21.7 ± 1.8 years old. Those with ADHD reported the average age of diagnosis to be 13.7 ± 7.7 years old.

The Adult ADHD Self-Report scale (AASRS-v1.1) checklist questionnaire was completed by all participants prior to participation; this was undertaken to quantify their ADHD symptomology. The AASRS-v1.1 consists of 18 questions that are highly correlated to the diagnostic criteria set out by the DSM-IV [44] and are rated on a five-point Likert scale ranging from “never” to “very often” for each question. This screening tool has been noted as highly effective for predicting ADHD symptomology [45] and is broken up into two parts, part A (inattentiveness) and part B (hyperactive/impulsive). When participants respond with “sometimes”, “often”, or “very often”, it is highly suggestive of ADHD. This questionnaire was included as a part of the pre-participation screening to ensure that we did not inadvertently include any participants who may have unknowingly had ADHD in the “control” group and likewise that we did not include any in the ADHD group whose symptoms have resolved since their diagnosis. To note, no specific total is associated with a definitive ADHD diagnosis; however, we were able to quantify the score for each participant and group (ADHD vs. control). The ADHD group had an average score for part A of 15.0 ± 3.33 (Controls: 6.25 ± 3.41) and 25.7 ± 5.29 (Controls: 10.17 ± 6.91) for part B. Six participants reported that they were taking medication relating to ADHD at the time of participation. The medications reported included: Adderall, Concerta, and Vyvanse. All of the participants completed the Edinburgh Handedness Questionnaire to determine their hand dominance. The number of left-hand dominant participants was made similar between groups. Specifically, the ADHD group had one left, five right, and four ambidextrous participants, while the neurotypical control group had one left, 10 right, and one ambidextrous participant. An EEG safety checklist was completed to ensure that participants did not have a recent (past five years) history of epilepsy, concussion, stroke, or brain injury that could potentially affect the electrophysiological results or make the task unsafe for their participation.

### 2.2. Auditory Alone

A unisensory auditory stimulus was presented in front of the participant from speakers placed bilaterally from the computer screen. The stimulus was a female verbalisation of the word red, blue, or green (duration ~250 ms, ~75 dB). This stimulus choice was due to the importance of semantic congruence between sensory cues in multisensory conditions (auditory and visual in this case) to enhance multisensory processing [17]. Auditory verbalisations have been established as effective at facilitating multisensory processing in previous literature utilising similar auditory cues [17,46,47,48].

### 2.3. Visual Alone

A unisensory visual stimulus consisted of a circle (diameter 300 mm, seated ~23 inches away from the screen) filled with the colour red, blue, or green. The circle was on a black background and lasted for a duration of 250 ms.

### 2.4. Audiovisual Multisensory

**Figure 1:**
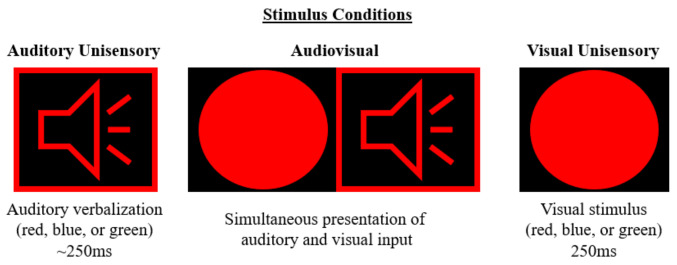
Visual depiction of the three unique stimulus conditions. Each condition was representative of the colour red, blue, or green. The audiovisual condition was always semantically congruent.

The multisensory stimulus consisted of the described auditory and visual conditions occurring simultaneously. The auditory and visual components were always semantically congruent. For example, when the blue circle appeared, the “blue” verbalisation was emitted, etc. The conditions were never semantically incongruent. Please refer to Figure 1 which depicts each sensory condition. 

To measure MSI, a two-alternative forced-choice discrimination task was utilised that consisted of three unique stimuli that were semantically congruent. For example, a female verbalisation of the word “red” was paired with a red circle, or verbalisation of the word “blue” was paired with a blue circle. Similar paradigms have been used previously to quantify multisensory processing [17,31,48,49]. This paradigm emphasises both response time and accuracy. E-Prime 2.0 Professional Software (Psychology Software Tools, Sharpsburg, PA, USA). was used to develop and implement this paradigm. A previous study had described the EEG and behavioural results, with an emphasis on response time (ms) and accuracy (%) while assessing neural markers in the form of ERPs [31]; thus, limiting the assessment of cortical activity to surface electrodes. Participants completed the task in two blocks, each being approximately 15–18 min long, with each block consisting of 110 auditory unisensory, 110 visual unisensory, and 110 multisensory conditions, totalling 330 stimuli per block. Each stimulus was preceded by a fixation cross in the centre of the black screen, which was utilised as an attentional cue to promote continued attentional allocation to the paradigm prior to each stimulus. The conditions were presented in random order with an equal probability of occurrence, with an inter-stimulus interval randomised within E-Prime and had a duration between 1000 and 3000 ms, thus reducing anticipatory processes. Continuous whole-head EEG was collected throughout the completion of this response paradigm. Further details on the paradigm used are outlined in McCracken et al., 2020 [31]. The current study aims to use source localization via sLORETA to assess the neural generators involved during this task in a population of adults with ADHD compared to neurotypical controls.

A Waveguard™ 64-electrode EEG cap (ANT Neuro, Hengelo, The Netherlands) was used to collect surface brain electrical activity in response to performing the forced-choice tasks in each of the three sensory conditions. The Waveguard™ cap was connected to a TMSi REFA-8 amplifier (TMSi, Oldenzaal, The Netherlands) with 64 EEG channels, four bipolar channels, and four auxiliary channels and was collected through Advanced Source Analysis Lab™ (ANT Neuro) at a sampling frequency of 2048 Hz. The EEG data were processed offline using ANT 4.10.1. The artifacts resulting from muscle activity and/or blinking were removed. A band-pass filter was utilised, with a low cut-off of 1.6 Hz to remove the constant slow-wave activity and a high cut-off of 45 Hz to remove any artifacts from the surrounding electrical equipment; this was performed with a slope of 24 db/octave, which was applied to individual data sets. Artifact rejection was performed by excluding waveforms that exceeded ±100 μV. The electrodes still containing significant noise (e.g., electrical contamination not related to the EEG signal) were interpolated for the relevant individual participant using the nearest surrounding eight electrodes. This was undertaken on an individual data set basis. The EEG data were then averaged per condition into 600 ms epochs (−100 to 500 ms) surrounding stimulus onset, giving three averages for each participant (auditory, visual, and multisensory). Current source analyses were performed on the entire epoch, meaning they were examined from −100 to 500 ms post stimulus onset, although with a particular interest within the 0–250 ms latency window, as previous multisensory work suggests early integration is specific to latencies prior to 250 ms post stimulus.

The primary objective of the research reported here was to build upon prior novel findings [31], and was, therefore, to assess whether there were differences in neural generators when those with ADHD were presented with multisensory and the constituent unisensory conditions when compared to neurotypical controls. Previous work was limited to the assessment of surface electrodes; however, the inclusion of source localization techniques in the present study allows for the determination of particular neural generators and activity that are outside of the scope of surface EEG by itself.

### 2.5. Data Analysis

#### 2.5.1. Source Localization—SLORETA Analysis

Source localization was performed to assess the neural areas of greatest activity in response to each sensory condition, allowing for a comparison between groups. This was performed using standardised low-resolution brain electromagnetic tomography (sLORETA) software [45]. sLORETA is a source analysis software that localizes neural generators. This is undertaken based upon covariance and statistical techniques, which include independent component analysis (ICA) and MRI localization [43]. Additionally, this utilises scalp-recorded electrical potential distributions using high-density EEG, which is used to compute the cortical three-dimensional (3D) distribution of current density [43]. The previous version, LORETA, has been validated by methods including fMRI [49], structural MRI [50], and positron emission tomography (PET) [51]. sLORETA solves the inverse problem via the assumption of synchronous and simultaneous activation of neighbouring neurons without a localization bias [45]. sLORETA has been validated for accuracy by comparing results with techniques such as EEG and fMRI [49,52]. The evidence from this validation research suggests that the estimated localized sources are reliable. The source localization analysis was completed in the time-domain to assess differences in areas of greatest neural activity between those in the ADHD group and those in the neurotypical control group in response to the: (1) multisensory, (2) visual unisensory, (3) and auditory unisensory conditions. The sLORETA settings divide cortical grey matter into 6239 voxels with a spatial resolution of 5 mm. Voxel-wise randomisation tests with 5000 permutations based on statistical nonparametric mapping (SnPM) were performed. This corrects for multiple comparisons and has the highest possible statistical power [53]. The standardised current density at each voxel is calculated using a head model and electrode coordinates based on the Montreal Neurological Institute (MNI) average MRI brain map (MNI-152).

#### 2.5.2. Time-Domain Statistical Analysis

The statistical significance was set to *p* = 0.05. The statistical analysis was conducted within sLORETA’s built-in statistical tool [43,54]. sLORETA completes an independent two-tailed Student’s *t*-test, converting collected EEG data into *t*-values for each time frame, comparing two independent groups, in this case, ADHD vs. controls. This was performed for 1229 time frames, which is 600 ms with a sampling frequency of 2048 Hz. This was performed using SnPM, which adjusts for multiple comparisons using Fisher’s random permutation test with 5000 randomisations [53]. The software sets a two-tailed *t*-value threshold, providing the t-critical, where once a value in the time-domain exceeds this threshold, a computation occurs within the software that localizes the area responsible for this neural activity while also providing the associated statistical significance (*p*-value). Additionally, ASA Lab™ (ANT Neuro) sLORETA tool was used to create graphics of each group’s neural activity during the timeframe that was established using the sLORETA analysis software for all sensory conditions. Graphics of localized activity were created and have been included for each condition (auditory, visual, and audiovisual) and each group. 

## 3. Results

### 3.1. Multisensory

**Figure 2:**
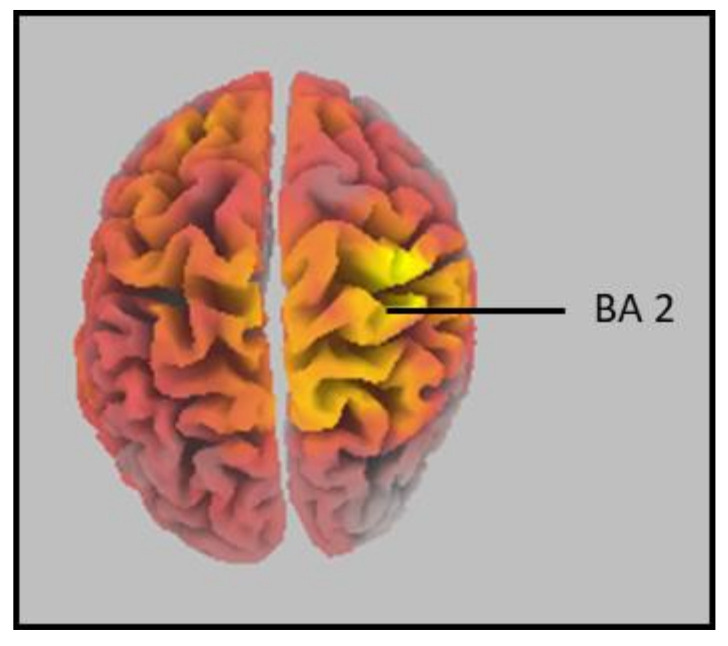
sLORETA 3D Cortex. Area highlighted indicates the region of maximal difference between groups (Control vs. ADHD), which was identified as right-hemispheric BA 2, postcentral gyrus, parietal lobe.

**Figure 3:**
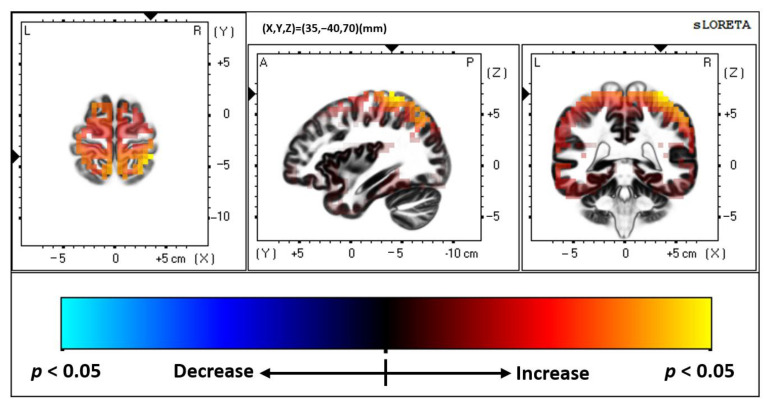
sLORETA multisensory response, ADHD vs. Controls. Slice Viewer highlighting region of greatest neural activity difference between controls and those with ADHD. Views, from left to right, include a transverse, sagittal, and coronal cross-sectional area. sLORETA analyses found that controls had greater activity in the region highlighted in yellow.

**Figure 4:**
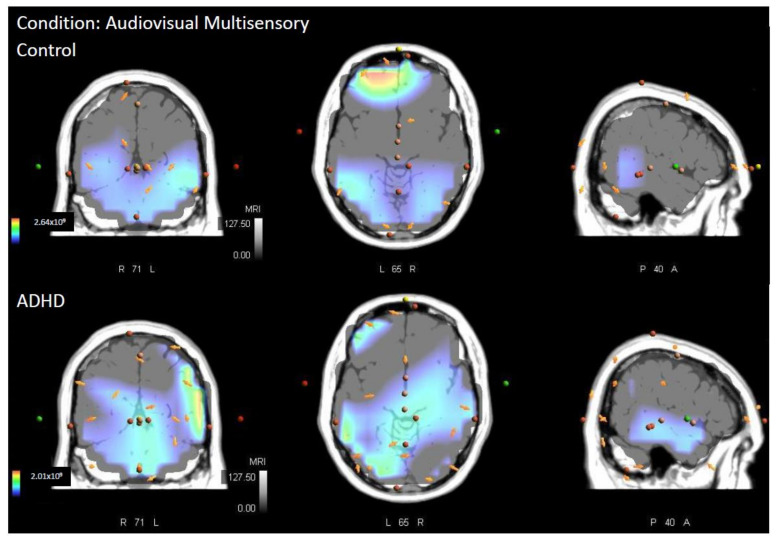
Average sources of neural activity for the audiovisual multisensory for each group (ADHD and Control) at 164 ms post stimulus onset. From left to right is the coronal (**left**), transverse (**middle**), and sagittal plane (**right**).

The comparison of multisensory responses between the ADHD group and the controls concluded that neural generators differed significantly between groups when presented with an audiovisual multisensory stimulus. The area of greatest difference in neural activity between groups was right hemispheric, Brodmann area (BA) 2, postcentral gyrus, parietal lobe (MNI coordinates: X = 35, Y = −40, Z = 70; *p* = 0.048). This can be seen in Figure 2 and Figure 3. The controls had significantly greater activity in this region at 164 ms post stimulus onset when compared to those in the ADHD group. Individual group activity can be seen in Figure 4. 

### 3.2. Visual Unisensory

**Figure 5:**
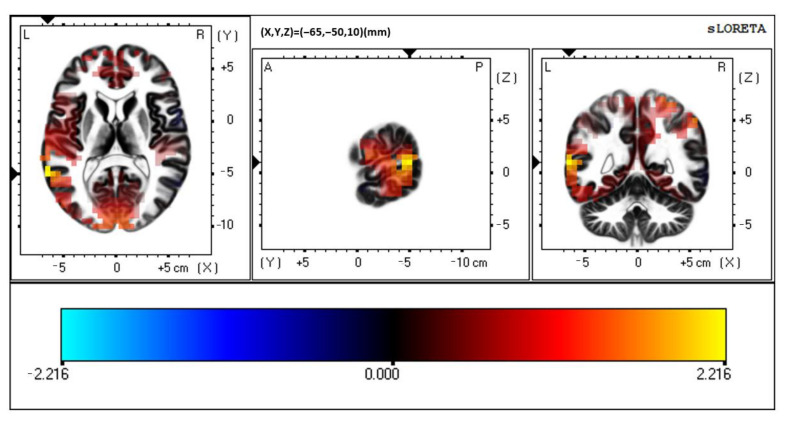
sLORETA visual unisensory response, ADHD vs. Controls. Slice Viewer highlighting region of greatest neural activity difference between controls and those with ADHD. Views, from left to right, include a transverse, sagittal, and coronal cross-sectional area. Control > ADHD activity at BA 22, left-hemispheric temporal lobe.

There were no significant differences between groups when presented with the visual unisensory condition (*p* > 0.05). No timeframe surpassed *t*-critical. However, although not meeting statistical significance, the area of greatest difference between groups when presented with the visual unisensory condition occurred at BA 22, superior temporal gyrus, left-hemispheric temporal lobe (MNI coordinates: X = −65, Y = −50, Z = 10; *p* = 0.182). Please see Figure 5. Controls activity > ADHD activity in this region at 165 ms post visual stimulus onset.

### 3.3. Auditory Unisensory

**Figure 6:**
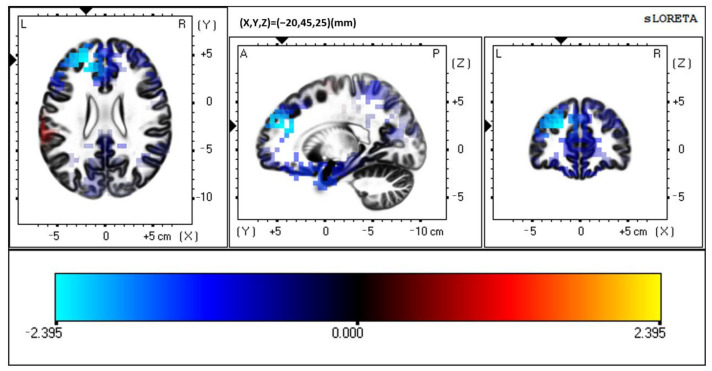
sLORETA auditory unisensory response, ADHD vs. Controls. Slice Viewer highlighting region of greatest neural activity difference between controls and those with ADHD. Views, from left to right, include a transverse, sagittal, and coronal cross-sectional area. ADHD > control activity at BA 10, left-hemispheric frontal lobe.

**Figure 7:**
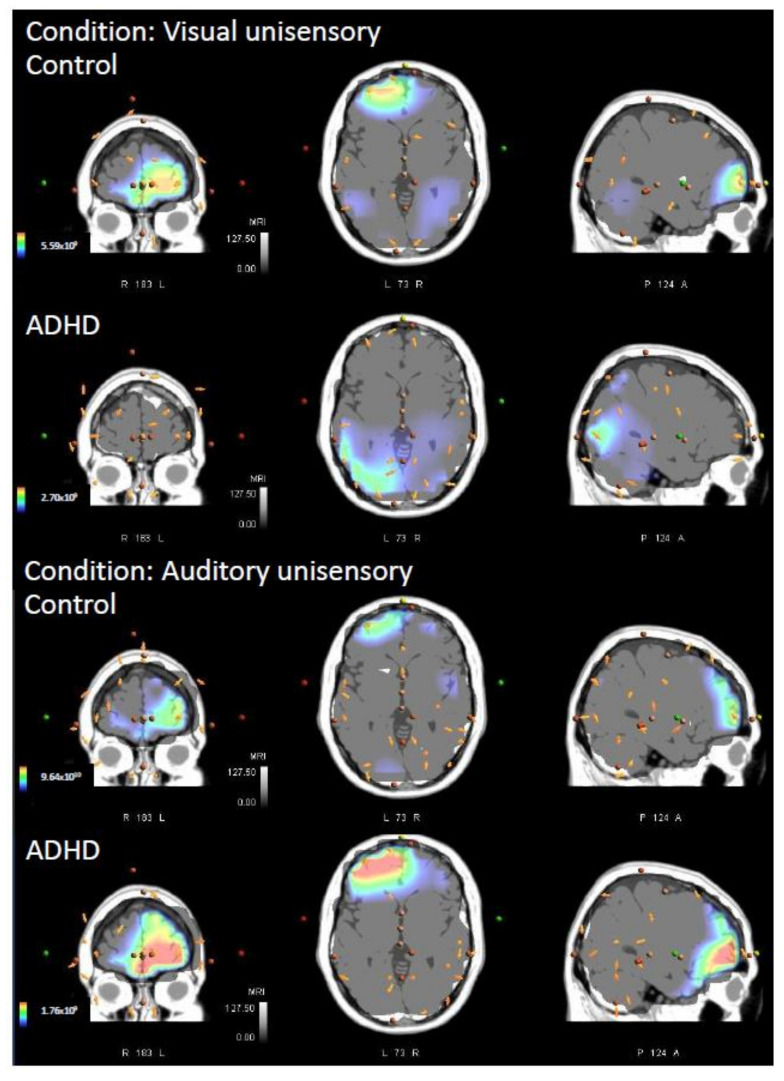
Average sources of neural activity for both unisensory conditions (top: visual, 165 post stimulus onset; bottom: auditory, 96 ms post stimulus onset) and for each group (ADHD and Control). Cross-sectional areas from left to right are coronal (**left**), transverse (**middle**), and the sagittal plane (**right**).

There were no significant differences between groups when presented with the auditory unisensory condition (*p* > 0.05). No timeframe surpassed *t*-critical. However, although statistical significance was not met, the auditory unisensory condition resulted in an area of greatest difference in BA 10, superior frontal gyrus, left-hemispheric frontal lobe (MNI coordinates: X = −20, Y = 45, Z = 25; *p* = 0.083). Please see Figure 6. ADHD activity > control activity in this region, at a latency of 96 ms post auditory stimulus onset. Each groups unisensory activity can be seen in Figure 7.

## 4. Discussion

This is the first study, to our knowledge, to assess the presence of unique neural generators, via source localization, related to multisensory processing in those with ADHD. Using source localization techniques and EEG data collected during an audiovisual multisensory task, we were able to compare cortical regions that differed in activation between young adults with ADHD and adult neurotypical controls. This additional analysis was an important next step from our previous work that assessed surface brain activity in those with ADHD during AV multisensory tasks [31,32]. The previous work was conducted using a 64-electrode whole-head EEG system, and the analysis was based upon the principle of superposition of electrical fields [30]. Therefore, improving spatial resolution via source localization was important. Therefore, the current sLORETA analysis was performed on the evoked potential data from those in McCracken et al., 2020 [31]. This allows for the assessment of specific neural structures, including those that are deeper to the brain surface and therefore outside the scope of a direct surface assessment. In the current study, those with ADHD were found to have unique neural activation in response to an audiovisual multisensory stimulus when compared to controls. BA 2 had reduced activation in those with ADHD when compared to controls, and this unique activation occurred in the right-hemispheric parietal lobe at 164 ms post stimulus onset. When comparing the neural response to each of the unisensory components, there were no significant differences between those with and without ADHD. This suggests that the difference discovered in BA 2 is unique to a multisensory response related to altered neural function in ADHD.

### 4.1. Brodmann Area (BA) 2

The primary somatosensory cortex (S1) has a fundamental role in processing afferent sensory input, particularly somatosensory input [55]. Additionally, S1 allows for the integration of both afferent and efferent signals, making it a strong contributor to the processes necessary for movement [55]. The primary somatosensory cortex encompasses BA 3, 1, and 2. All of these areas are highly involved in motor learning, the localization of touch, and sensory perception [56]. Each BA has a specific function that are slightly different to the others. Specifically, BA 3 is involved in processing vibration, pressure, and general tactile stimuli; BA 2 is involved in pressure and joint position sense; whereas BA 1 generally responds to vibrotactile stimuli [57]; each area is reciprocally connected. Lesions in any of these BA would result in alterations to proprioception and fine touch due to the involvement of the dorsal column medial lemniscus (DCML) pathway [56]. More specifically, BA 2 is postulated to respond to pressure, joint position, and complex touch [57,58]. The results from the current work suggest that BA 2 functions differently in those with ADHD when responding to multisensory afferent input. 

The right-hemispheric difference specific to BA 2 is consistent with our previous work on ADHD. Specifically, we previously reported that when presented with an AV multisensory stimulus, neurotypical controls had a more pronounced negative ERP over right-hemispheric centro-parietal brain regions, specifically electrodes CP4 and P6, when compared to young adults with ADHD [31]. This prominent negative activity present in controls, but not those with ADHD, was evident from 160–180 ms post-stimulus onset [31]. The activity found in our previous work was in a similar location, and within the same time frame, as that of the results from the current study using source localization techniques analysing the same participants’ EEG data.

Additionally, the previous analysis yielded results showing that those with ADHD had greater activity in areas related to multisensory integration in parietal-occipital brain regions (electrodes PO7, PO8, O1, and O2) at an earlier latency, from 110 to 130 ms [31]. Altered activity in BA 2 in ADHD may be related to differences in the way those with ADHD process and respond to multisensory stimuli, as BA 2, as a result of the involvement of the DCML pathway, has a primary role in somatosensation such as mechanoreceptive sensation and proprioception, receiving input from joints and deep tissues [56,59,60,61]. For instance, one interpretation of this difference is an altered perception of the digits or limb used to respond to the stimulus. The multisensory condition resulted in greater activity in the controls in BA 2 compared to those with ADHD, and this may be related to the faster responses recorded in an ADHD group [31]. However, this is unlikely, as this unique neural difference was only present during the multisensory condition. Reduced activation may reflect more efficient processing and thus be associated with the quicker responses found previously. However, there may be other, more likely, postulated mechanisms involved in this differed neural activation, such as impaired attention resulting in suboptimal multisensory processing, and prioritising the speed of response over accuracy. This explanation will be further described in the subsequent section. 

### 4.2. Parietal Lobe

The current study detected altered activity within BA 2 specific to the right hemisphere in young adults with ADHD. BA 2 is found within the parietal lobe. The parietal lobe is generally associated with sensory processing and contains the primary (S1) and secondary (S2) somatosensory cortices [62]. Parietal deficits are also well-noted in ADHD [63,64]. Interestingly, a previous work noted that a reduction, or attenuation, in parietal ERPs were associated with reductions in performance [63]. Conversely, the opposite was found in adults, where those in the ADHD group had shorter response times than controls [31]. Furthermore, previous research suggests that the right parietal lobe and its function may be implicated in the neurophysiology of ADHD [65,66]. Neurophysiological evidence supports the role of altered right parietal lobe function in ADHD, including more left-sided errors than controls and reduced learning on right-parietal-dominant tasks [65]. These previous findings suggest alterations to functions related to the parietal lobe and ADHD, which likely are related to structural alterations as well. 

Anatomically, S1 is located at the postcentral gyrus of the parietal lobe, and an altered structure may have important functional implications and vice versa. Imaging studies have noted that children with ADHD have significantly reduced cortical volume in the right parietal lobe [67]. A previous study established that there is an AV multisensory effect focused over the right hemisphere at approximately 160 ms until 180 ms in neurotypical humans [30]. These regions and latency coincide with the results found in the current study, suggesting that both may have important functional implications for MSI. Thus, these changes established herein have relevance to those with ADHD and how their nervous system processes such multisensory cues.

An fMRI study with both clinical and behavioural importance found right-parietal dysfunction in young boys with ADHD [64]. Specifically, the controls had greater activation in the right parieto-occipital areas (BA 19) and right inferior parietal lobe (BA 40) compared to children with ADHD, further supporting the role of right parietal dysfunction in ADHD [64]. This evidence further supports the suggestion of right striatal-parietal dysfunction in adolescents with ADHD. The right parietal lobe has an attributed role in spatial attention, including right-hemisphere involvement of the fronto-parietal network. Additionally, spatial attention has been localized to the right-hemispheric fronto-parietal networks [64,66,68,69,70]. Alterations to the right hemisphere parietal activation are associated with clinical outcomes in both adolescents and children, such as impaired control of attention [70]. This was observed as children with ADHD have an impaired ability to attentionally orient to the left visual field [71]. Alternatively, children with ADHD were found to have under-activation in the right SPL during a visual selective attention task [72]. This suggests that well-noted parietal dysfunction is present in both children and adolescents with ADHD. Attentional alterations are also present and characteristic of ADHD, and therefore the previously noted alterations to attentional networks and the findings from the current study localized to BA 2 suggest that right-parietal alterations impact multisensory processing and relate to the symptomology of ADHD.

Those with ADHD responded differently at a neural level in response to the multisensory condition; this unique difference in those with ADHD was found in BA 2, the parietal lobe. It should be noted that this difference was unique to the multisensory condition, and no group differences were found in either unisensory condition. This further suggests that the unique neural activation is a result of the multisensory nature of the task. One potential explanation for these differences in sensory perception between the groups could be that those with ADHD-like traits have a reduced ability to discern when multiple stimuli occur simultaneously [24]. This is described as having an altered temporal integration window and may have important implications for the perception of multisensory inputs as a result of alterations to attentional capabilities, leading to increased distractibility [24]. For multisensory inputs to be processed and integrated as multisensory, and not two unique individual stimuli, it is necessary for each of the constituent components of the target stimulus to be attended to. In the case of the current study, individuals needed to attend to and process both auditory and visual unisensory afferents simultaneously. It is possible that the difference found within right-hemisphere BA 2, parietal lobe, is a result of altered attentional capabilities in those with ADHD. Thus, potentially reducing the activity within the parietal lobe in the ADHD group as a result of their inability to allocate attentional resources to each of the multisensory components. This suggests that the structural alterations in parietal regions may have significant implications for how those with ADHD process sensory information and consequently respond in multisensory environments. Thus, it has important implications for their sensory perception and how they experience everyday environments full of audiovisual stimuli.

### 4.3. Multisensory Processing

Although limited literature has assessed MSI in ADHD, previous work has shown that there are differences in the way that adults with ADHD process and respond to multisensory inputs at both the behavioural and neurological levels. MSI is fundamental to how individuals experience the world, as many environments are multisensory in nature. For instance, at any given time, individuals are presented with auditory, visual, and tactile stimuli. Therefore, alterations to multisensory processing may have fundamental implications for how one processes, perceives, and responds to their environment, potentially impacting day-to-day activities such as working and socialising. These differences can be observed using neurophysiological and behavioural measures. For instance, historically, S1 was often thought of as unisensory but has more recently been associated with being the initial site for processing associated with MSI [55,73]. Therefore, neural changes in the right-hemisphere S1 associated with ADHD [74] may have important implications for MSI.

The previous unique neural alterations relating to MSI in ADHD were predominantly localized to parietal, occipital, and central electrode regions [31,32]. These changes were found in conjunction with behavioural alterations, such as significantly shorter response times. Additionally, although there was not a significant effect found for accuracy, those with ADHD showed a pattern of increased error compared to controls, and a medium effect size was present [31]. These results provide further insight into the neural mechanisms that may be related to these behavioural findings. The current work found that those in the ADHD group exhibited attenuated activity within the right-hemisphere parietal lobe. As previously discussed, this region is highly involved in attentional capacity [70]. Therefore, the behavioural differences noted in prior research may be a result of or related to reductions in attention in ADHD as a result of right parietal dysfunction. This indicates the possibility of attenuated neural activity being related to impaired attention during the multisensory condition, potentially associated with important behavioural findings, such as increased error, although with shorter responses.

A tenet of multisensory processing that can have a profound influence on the integration effect is related to the allocation of attentional demands to each sensory component that encompasses the multisensory stimulus. This suggests that the level of selective attention can modulate MSI [75]. For instance, allocating attention to both the auditory and visual components of a multisensory condition can have a critical effect on the electrophysiological responses typically associated with MSI [75]. This is observed as the absence of, or reversal of, the neural enhancements associated with MSI when attention is limited to one of the constituent stimuli components. Therefore, reductions in attention may result in multisensory dysfunction, and this could have important implications for understanding the neural mechanisms involved in multisensory processing in those with ADHD, such as those found in the current study. Furthermore, a previous source localization study found hypoactivation within the right-hemispheric frontal gyrus (BA 32) in response to incongruent multisensory inputs during incompatible NoGo trials in a Go/NoGo task in adolescents with ADHD when compared to neurotypical controls [23]. The authors postulated that this was related to compromised response inhibition as a result of impulsivity [23]. These findings complement those found in the current study, suggesting a reduced activation in the right-hemispheric regions during multisensory conditions and that this could be associated with impaired performance measures, such as accuracy. Additionally, the activation differences relating to S1 suggest relevance to other neural processes, as S1 has a primary role in many functions, including but not limited to those involved in sensorimotor integration in relation to motor learning. This suggests that distractibility and inattention, and their relation to learning and performance, may be impacted in ADHD.

### 4.4. Limitations

This study focused on university-aged individuals recruited from the university campus. Thus, limiting the generalizability of findings to those outside of this age group, such as children or adolescents with ADHD. A limitation of the current work is the utilisation of the entire group of adults with ADHD and that it does not specify or compare by subtype (i.e., inattentive vs. hyperactive). In the future, work should address this and explore potential differences in multisensory processing between ADHD subtypes. Additionally, although utilising sLORETA software provides a strong basis for the interpretation of neural generators, it should be noted that this involves pairing collected EEG data with a standardised average MRI. Therefore, although this is an acceptable, validated, and cost-effective technique to interpret neural activity, the MRI used for localization is not definitively reflective of each individual’s specific neural structures. In the future, incorporating fMRI techniques and individual scans would enhance the findings noted above.

## 5. Conclusions

The present data analysis yielded results indicating that young adults with ADHD process AV multisensory stimuli differently than controls. In particular, neural differences were localized to the right hemisphere over parietal regions associated with BA 2. No statistically significant differences in neural generator activity between groups were found for either unisensory condition. Therefore, the differences noted herein are unique to multisensory processing in those with ADHD. This result may have important implications for how they process and respond to multisensory inputs while also reflecting alterations to attentional capacity when more than one stimulus is presented at a time. Our previous work utilising the same data found that neurotypical controls had greater activity in this brain region, specifically over right-hemispheric centro-parietal (CP4 and P6 electrodes) brain regions from 160 to 180 ms [31], suggesting a similar, yet more specific, pattern of activity yielded using sLORETA analysis techniques. This reflects the utility of source localization techniques to further elaborate on EEG findings in this population. In the future, continuing this stream of work via the utilisation of fMRI could prove beneficial. Overall, this work suggests that multisensory tasks and EEG in conjunction with source localization techniques may have the potential to serve as an objective measure of altered MSI in those with ADHD. Furthermore, the activity alterations localized over the right hemisphere in S1 indicate that other important alterations, such as those relating to attentional impairments, may be present in young adults with ADHD and have important implications for many functions, including motor learning and sensory integration.

## Data Availability

The data presented in this study are available in Figure 2, Figure 3, Figure 4, Figure 5, Figure 6 and Figure 7, and spreadsheets can be made available upon request.
